# Sympathetic-correlated c-Fos expression in the neonatal rat spinal cord in vitro

**DOI:** 10.1186/1423-0127-16-44

**Published:** 2009-05-01

**Authors:** Chun-Kuei Su, Chiu-Ming Ho, Hsiao-Hui Kuo, Yu-Chuan Wen, Chok-Yung Chai

**Affiliations:** 1 Institute of Biomedical Sciences, Academia Sinica, Taipei 115, Taiwan, Republic of China; 2 Department of Anesthesiology, Taipei Veterans General Hospital and National Yang-Ming University, Taipei 112, Taiwan, Republic of China; 3 Department of Physiology and Biophysics, National Defense Medical Center, Taipei 114, Taiwan, Republic of China

## Abstract

An isolated thoracic spinal cord of the neonatal rat in vitro spontaneously generates sympathetic nerve discharge (SND) at ~25°C, but it fails in SND genesis at ≤ 10°C. Basal levels of the c-Fos expression in the spinal cords incubated at ≤ 10°C and ~25°C were compared to determine the anatomical substrates that might participate in SND genesis. Cells that exhibited c-Fos immunoreactivity were virtually absent in the spinal cords incubated at ≤ 10°C. However, in the spinal cords incubated at ~25°C, c-Fos-positive cells were found in the dorsal laminae, the white matter, lamina X, and the intermediolateral cell column (IML). Cell identities were verified by double labeling of c-Fos with neuron-specific nuclear protein (NeuN), glial fibrillary acidic protein (GFAP), or choline acetyltransferase (ChAT). The c-Fos-positive cells distributed in the white matter and lamina X were NeuN-negative or GFAP-positive and were glial cells. Endogenously active neurons showing c-Fos and NeuN double labeling were scattered in the dorsal laminae and concentrated in the IML. Double labeling of c-Fos and ChAT confirmed the presence of active sympathetic preganglionic neurons (SPNs) in the IML. Suppression of SND genesis by tetrodotoxin (TTX) or mecamylamine (MECA, nicotinic receptor blocker) almost abolished c-Fos expression in dorsal laminae, but only mildly affected c-Fos expression in the SPNs. Therefore, c-Fos expression in some SPNs does not require synaptic activation. Our results suggest that spinal SND genesis is initiated from some spontaneously active SPNs, which are capable of TTX- or MECA-resistant c-Fos expression.

## Background

Autonomous generation of tonic sympathetic activity is fundamental to normal visceral functions. The primary source for sympathetic tone generation is the brain stem [1-4]. However, in neonatal rats, three or fewer thoracic spinal cord segments contain sufficient neural elements for spontaneous generation of sympathetic nerve discharge (SND; [5]). In vitro studies have reported that some sympathetic preganglionic neurons (SPNs) are spontaneously active in the absence of extraspinal inputs [6-9]. These in vitro observations are consistent with the in vivo observations that, being deprived of supraspinal inputs, isolated spinal cords generate substantial amounts of SND in adult animals [10-14]. Although an isolated spinal cord could generate SND under certain pathophysiological conditions, the spinal neurons responsible for SND genesis were largely unknown.

Findings obtained from the studies of neural elements involved in sympathetic regulation at the level of the spinal cord could provide clues to elucidate the anatomical substrates underlying spinal SND genesis. It was found that peripheral visceral afferents project to the gray matter of the spinal cord at both superficial (lamina I and II) and deep layers (laminae V, VII, and X) [15-17], wherein sympathetic interneurons are concentrated in the deeper layers [18-20] and scattered in more dorsal laminae [21,22]. Moreover, a group of GABAergic sympathetic interneurons is located in the central gray matter or lamina X [23]. All these studies suggest that sympathetic-correlated neurons are mainly distributed in medial dorsal parts of the spinal cord.

Using a reduced preparation that only retained splanchnic sympathetic nerve-thoracic spinal cord, we demonstrated that one prerequisite condition for in vitro SND genesis is to incubate the spinal cord at an ambient temperature ≥ 20°C [24]. The optimal temperature for the cord to generate SND in the splanchnic nerves is 24.5 ± 1°C [5,24]. However, because activities of different sympathetic nerves originate in nonoverlapping spinal segments [5,25,26], whether the thoracic cord in vitro can generate SNDs in other sympathetic nerves was not clear.

The expression of c-Fos protein is often used as a marker of neural activity [27]. Different experimental paradigms have been used to stimulate c-Fos expression in the central nervous system to elucidate the involved anatomical substrates [28-35]. In this study, we first established that cervical and splanchnic sympathetic nerves share a common temperature-dependent scheme for SND genesis. We then incubated the spinal cord at ambient temperatures favorable or unfavorable for SND genesis, and explored the resulting c-Fos expression patterns. Manipulations of c-Fos expression patterns were achieved by applications of tetrodotoxin (TTX) to block action potential generation, or applications of mecamylamine (MECA) to exert a broad-spectrum blockade of nicotinic receptor activities that would attenuate SND [36]. For comparisons, a negative control of drug effects on c-Fos expression was achieved by applications of kynurenate (KYN), which did not suppress SND genesis in the cord [37]. By determining SND-correlated c-Fos expression patterns, we aimed to elucidate the anatomical substrates underlying SND genesis.

## Materials and methods

### General procedures

Neonatal Sprague Dawley rats were used in this study (1–6 days old; *n *= 99). All protocols were approved by the Institutional Animal Care and Utilization Committee of Academia Sinica (Protocol#: PRaIBMSC2003014). Surgical procedures were as previously described [24]. Briefly, halothane-anesthetized neonates were promptly decerebrated and transcardially perfused with ~4°C artificial cerebrospinal fluid (aCSF; in mM: 128 NaCl, 3 KCl, 1.5 CaCl_2_, 1.0 MgSO4, 24 NaHCO_3_, 0.5 NaH_2_PO_4_, 30 D-glucose, and 3 ascorbate; equilibrated with 95% O_2_-5% CO_2_). After dissection, thoracic spinal cord segments (T1-T12) were retained. The cervical sympathetic nerve was prepared by freeing a stub of the right paravertebral sympathetic trunk from surrounding connective tissue at the level of the first and second ribs. The splanchnic nerve, mainly containing preganglionic fibers, was easily identified by its innervation to the celiac ganglion located adjacent to the adrenal gland.

### Neural recordings and signal analysis

The nerve-cord preparation was kept in a thermo-controlled bath chamber containing 30 ml freshly oxygenated aCSF. Compound action potentials generated from the cervical and splanchnic sympathetic nerves were recorded using suction electrodes. Neural signals were amplified, filtered at a bandpass of 0.1–1 kHz (DAM50; World Precision Instruments, Sarasota, Florida, USA), and stored on a tape recorder (Neuro-Corder DR-890; CygnusTechnology Inc., Delaware Water Gap, Pennsylvania, USA). The processing of signals followed methods previously described [5]. Briefly, SND signals were integrated using a custom-made leaky integrator (discharging time constant: 15 ms) to display the SND envelope. For quantitative evaluation of its oscillating patterns, signals of the SND envelope were digitized (Digidata 1322A; Axon Instruments; Molecular Devices Corp, Sunnyvale, California, USA), and sampled at 0.4 kHz to obtain 16 data segments with each registering 40.96 s. The power spectra of the SND envelope were constructed using Axograph (version 4.9; Axon Instruments). The spectral components that are truly attributed to neural activity were extracted by subtracting from the spectra of background noise, which was determined by adding 100 mM KCl into bath solution for depolarization-blockade of action potential generation. Power spectra of each experiment were normalized to their peak power at ~1 Hz, which allowed data pooling from individual experiments to acquire average power spectra.

### Drugs and drug application

Drugs were purchased from Sigma (St. Louis, Missouri, USA) and dissolved in water to prepare concentrated solutions. To avoid spontaneous SND genesis that might lead to endogenous c-Fos synthesis at 25°C, drugs were applied by adding an aliquot of concentrated solutions directly to the bath solution when the cord was incubated at ≤ 10°C. Suppression of SND genesis was attained by application of 0.5 μM tetrodotoxin citrate (TTX) or application of 20 μM mecamylamine hydrochloride (MECA) to block endogenously active nicotinic receptors [36]. In some experiments, application of 800 μM kynurenate (KYN) was used for a broad-spectrum blockade of ionotropic glutamate receptors. After drug applications, the incubation temperature was taken up to 25°C to allow c-Fos synthesis for 3 h under in vitro conditions.

### Immunohistochemistry

After incubation under different experimental conditions, the spinal cords were immersed in 4% paraformaldehyde overnight and cryoprotected with 30% sucrose in 0.1 M phosphate buffer saline (pH 7.4). Frozen tissue blocks were routinely cut into transverse (T3/T6/T9) or horizontal sections (T7-T8) at 25–40 μm thicknesses. In some experiments, sections of other thoracic spinal cord segments were sampled for comparative purposes. Two series of immunohistochemical protocols, the diaminobenzidine (DAB) reaction or conjugated-fluorescent markers, were used to reveal c-Fos protein. Pretreatment of free-floating sections to quench endogenous peroxidase activity was performed following procedures as described in other studies [38-40]. The blocking of nonspecific staining and the enhancement of immunoreagent penetration were achieved by pretreating sections in 10% normal goat serum containing 0.3% Triton X-100. Sections were then incubated in affinity-purified rabbit anti-c-Fos polyclonal antiserum (1:3000; *sc*-52, Santa Cruz Biotechnology Inc, Santa Cruz, California, USA) for 48 h at 4°C, followed by incubation with the secondary antibody, biotinylated goat anti-rabbit IgG (1:150; Vector Laboratories Inc., Burlingame, California, USA) for 1 h at 4°C. Immunoreactive signals were further amplified by ABC solution (1:100; Vector Laboratories Inc.) and revealed by a nickel-intensified DAB reaction, with peroxide generated by glucose oxidase [41]. This was accomplished by incubating sections in the DAB-nickel-glucose-oxidase solution for 0.5–2 min. At the end of the DAB reaction, sections were washed and mounted onto gelatin-coated slides. In some experiments, sections were counter-stained with cresyl violet before being dehydrated in ethanol, cleared in xylene, and coverslipped.

Another series of tissue sections were prepared for the double labeling of c-Fos with choline acetyltransferase (ChAT), glial fibrillary acidic protein (GFAP), or neuron-specific nuclear protein (NeuN). Sections were incubated in a mixture of two primary antisera for 48–72 h at 4°C. The mixture of two primary antisera in 2% normal donkey serum contained affinity-purified rabbit anti-c-Fos polyclonal antiserum (1:4000; Santa Cruz Biotechnology Inc.) and affinity-purified goat anti-ChAT polyclonal antiserum (1:100; AB144P, Chemicon, Millipore Corp, Billerica, Massachussetts), mouse anti-GFAP monoclonal antiserum (1:1000; MAB360, Chemicon) or mouse anti-NeuN monoclonal antiserum (1:1000; MAB377, Chemicon). Specificity of the polyclonal primary antisera against c-Fos or ChAT for immunohistochemical staining in the rat spinal cord has been demonstrated in previous studies [35,42]. Fluorescent-marker-conjugated secondary antisera were purchased from Chemicon. Sections were incubated for 2 h in a diluted mixture (1:100) of donkey anti-rabbit IgG with donkey anti-goat or donkey anti-mouse IgG. After coverslipping, sections were imaged with a 10–40× or 60× oil-immersion objective on a confocal laser scanning microscope system (Bio-Rad Radiance 2100). Fluorescent markers, including fluorescein isothiocyanate (FITC) or rhodamine, were excited by a 488 or 543 nm laser beam and visualized via a HQ515/30 or E570LP emission filter. Some images were obtained by collapsing 8–16 consecutive 0.8 μm thick optical sections into a single image.

### Image data analysis

Terms and abbreviations of the histological structures of the cord were named according to the nomenclature described by Paxinos et al. [43]. The numbers of cells double-labeled for c-Fos and NeuN in the intermediolateral cell column (IML) of T3 spinal segments were counted manually. Counts only included the nuclear staining that aggregated in the lateral horn (see Results). For each experiment, the number of cells was averaged from counts of 5 sections, which were randomly selected from ~30 transverse sections. Data from different experiments were grouped according to treatments. The difference in the numbers of cells between groups was evaluated using one-way analysis of variance followed by group comparisons using the least significant difference method. A *P*-value of < 0.05 was considered significant. Values are presented as the mean ± SEM.

## Results

### Temperature-dependent SND genesis in thoracic spinal cord

**Figure 1:**
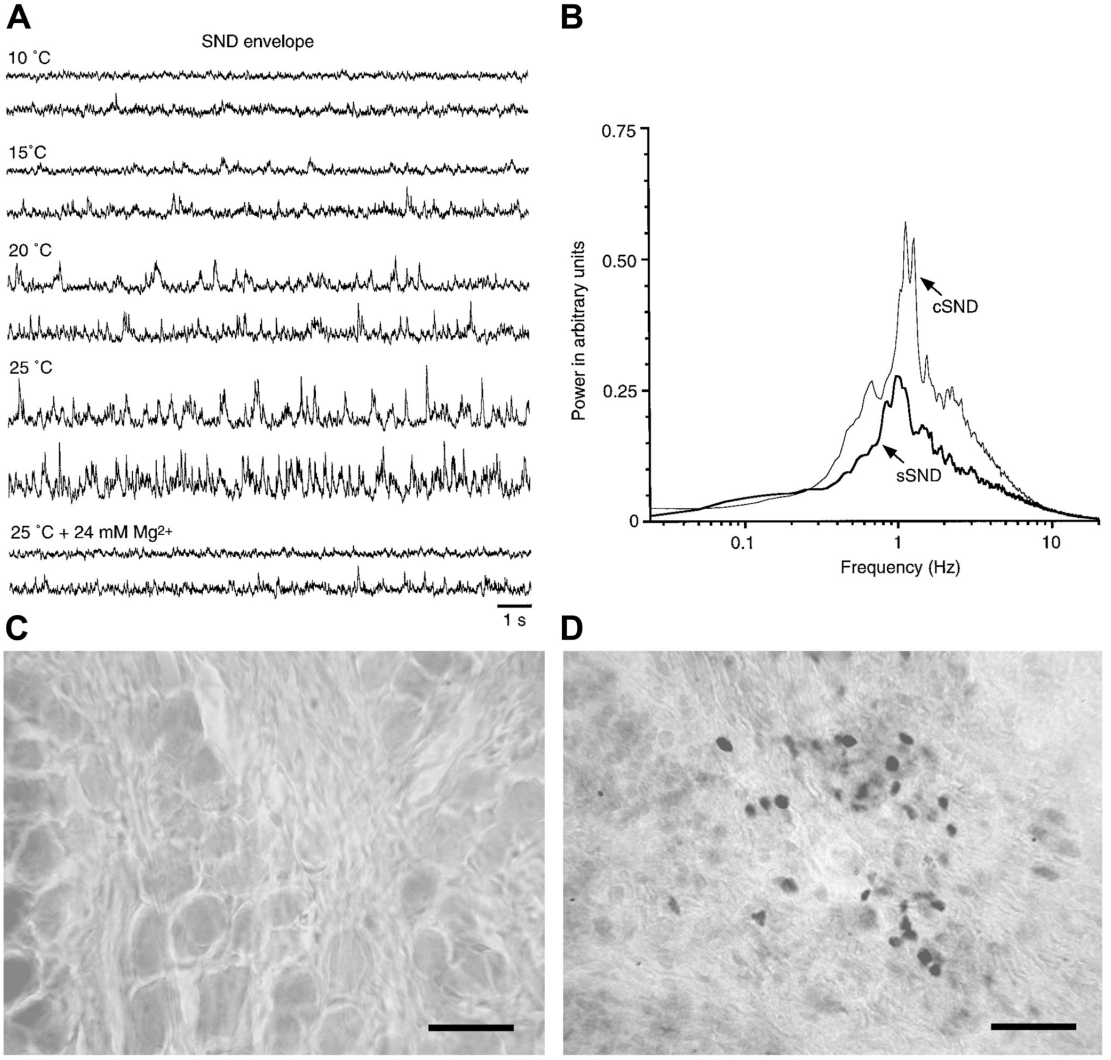
**Temperature-dependent features for in vitro nerve-cord preparations to generate sympathetic nerve discharge (SND)**. (A) Original traces of the SND envelope. Incubation temperature is indicated at the top of each panel. Upper and lower traces in each panel show envelopes of cervical SND (cSND) and splanchnic SND (sSND), respectively. SNDs were discernible at ≥ 20°C. Adding 24 mM Mg^2+ ^to the bath solution at 25°C reduced SNDs to the level at 10°C. (B) Power spectra of cSND and sSND envelopes at 25°C. Spectra were averaged from 21 experiments. Power spectra of both SNDs were similar, showing a peak at ~1 Hz. Photomicrographs in (C) and (D) show c-Fos protein expression in the dorsal root ganglion and celiac ganglion, respectively. The observation was obtained from a nerve-cord-ganglion preparation that incubated under optimal in vitro conditions for SND genesis (25°C normal aCSF, 3 h). c-Fos-positive cells were absent in the dorsal root ganglion (C), but present in the celiac sympathetic ganglion (D). Scale bars = 30 μm in (C, D).

Spontaneous generation of cervical SND (cSND) and splanchnic SND (sSND) was examined when thoracic spinal cords were incubated at different bath temperatures (*n *= 21). At ≤ 10°C, both SNDs were quiescent (Fig. 1A). At ≥ 20°C, SNDs were discernible and gradually augmented by increasing bath temperature. Oscillatory patterns of cSND and sSND were similar, showing power spectra with maximal peaks at ~1–2 Hz (Fig. 1B). At 25°C, spontaneous generation of SNDs persisted for ≥ 8 h. We therefore considered 25°C as the optimal temperature for SND genesis in vitro.

### Optimal incubation time for in vitro c-Fos expression

**Figure 2:**
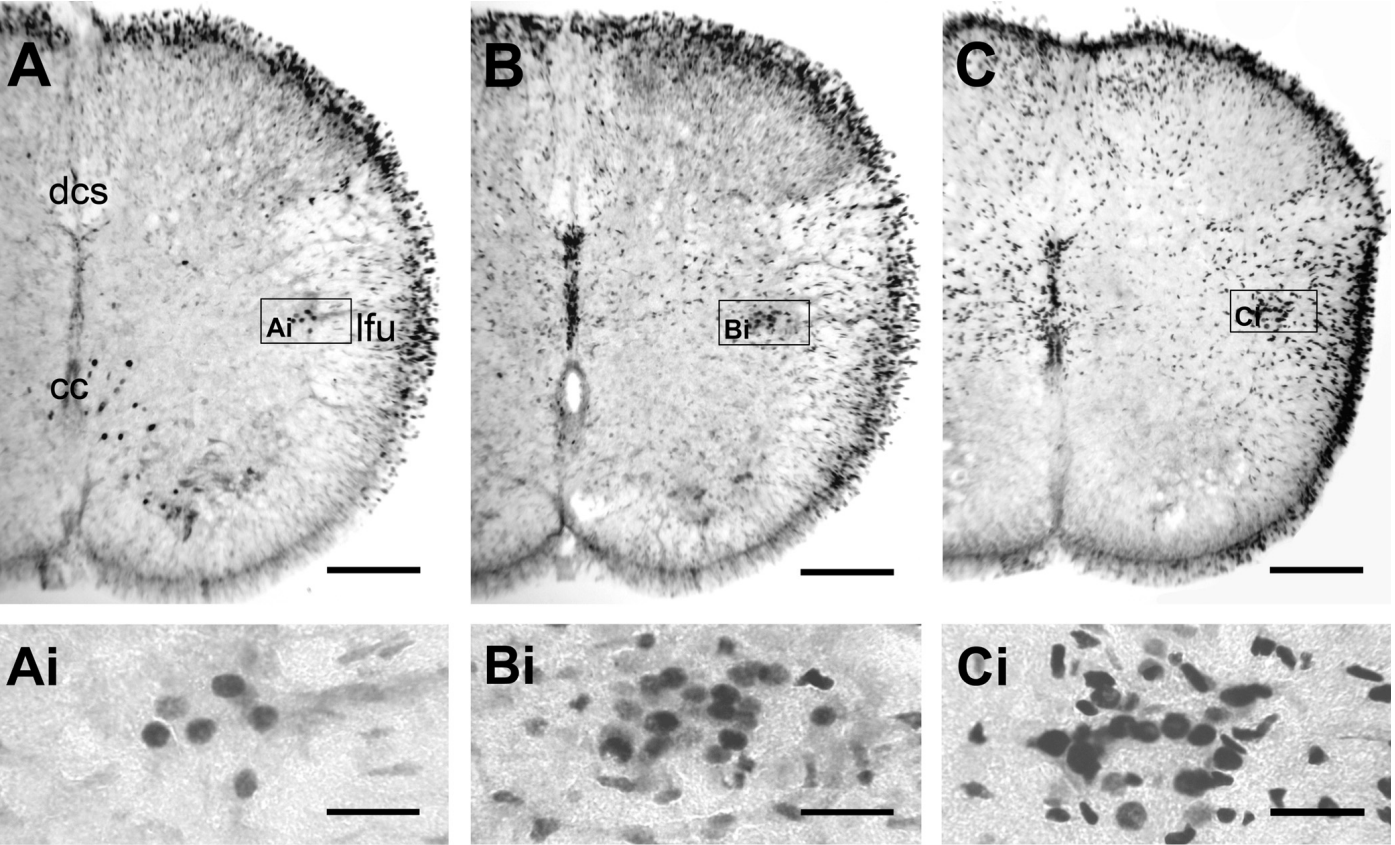
**Time course of c-Fos expression in the cords incubated in 25°C normal aCSF**. Photomicrographs show transverse sections of T3 from three preparations with different incubation time in hours: 1.5 (A), 3 (B), and 6 (C). After 1.5 h incubation, limited cells with intense c-Fos immunoreactivity (IR) were found in the IML (Ai). As the incubation time was prolonged to 3 h, c-Fos-positive cells in the IML increased (Bi), and were comparably abundant in the cord after the 6 h incubation (Ci). Note the presence of c-Fos-positive cells in the ventral horn and around the cc in (A) and the absence of these cells in (B, C). Also note the time-dependent spread of cell distribution to medial ventrolateral regions of the cord. Histological abbreviations in this and following figures: cc, central canal; dcs, dorsal corticospinal tract; IML, intermediolateral cell column; lfu, lateral funiculus. Scale bars = 150 μm in (A-C) and 30 μm in (Ai-Ci). Detailed distributions of c-Fos-positive cells in the cord are shown in following figures.

At the bath temperature optimal for SND genesis, the incubation time required for synthesis of detectable amounts of c-Fos was empirically evaluated in five preliminary experiments. Each experiment was constituted by three thoracic spinal cords incubated in normal aCSF for 1.5 h, 3 h, and 6 h. Figure 2 shows that oval or round nuclei with prominent c-Fos-immunoreactivity (IR) were concentrated in the IML of the cord incubated for 1.5 h. More c-Fos-positive nuclei were observed in the IML of cords incubated for 3 h. An extension of incubation time to 6 h resulted in a wider spread of c-Fos-positive nuclei in the medial ventrolateral regions. However, for cords with in vitro incubation times of 3 h and 6 h, similar c-Fos staining was observed in the IML. We therefore chose 3 h as the optimal incubation time for in vitro c-Fos expression.

To verify that c-Fos expression is intrinsic to the cord and not secondary to primary afferent activation, we examined c-Fos expression in the dorsal root ganglion and the celiac ganglion (*n *= 5). Experiments were performed in preparations that received intact primary afferents from the dorsal root ganglia and had their splanchnic nerves innervated the celiac ganglion. These nerve-cord-ganglia preparations were incubated at 25°C for 3 h. c-Fos-positive cells were absent in the dorsal root ganglia (Fig. 1C); however, they were present in the celiac ganglion (Fig. 1D).

### SND-correlated c-Fos expression in thoracic spinal cord in vitro

**Figure 3:**
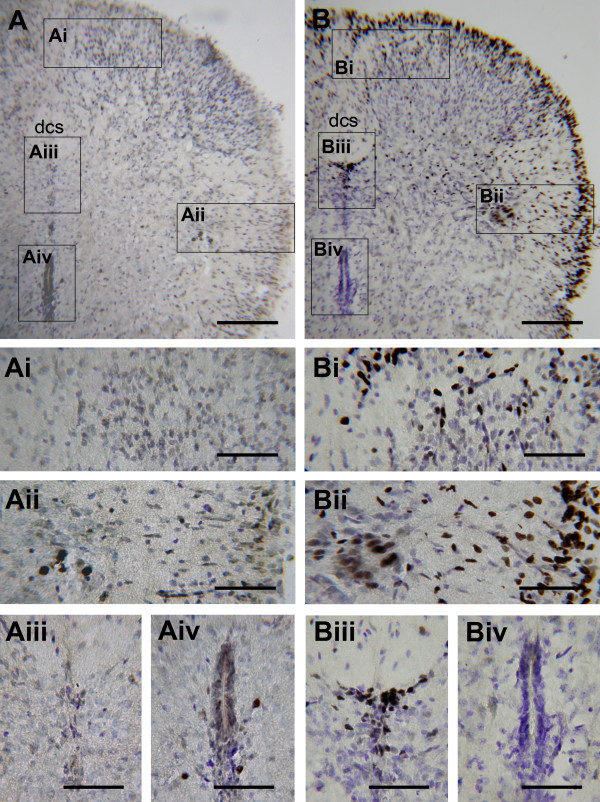
**Temperature-dependent in vitro c-Fos expression in the cord**. Tissue sections were counterstained with cresyl violet. Photomicrographs show the dorsal quadrant of transverse sections of T3 from the cord with 3 h incubation in ≤ 10°C (A) or 25°C (B) normal aCSF. In the cord incubated at ≤ 10°C, only a few c-Fos-positive cells were found in the IML (Aii) and around the cc (Aiv). In the cord incubated at 25°C, abundant c-Fos-positive cells concentrated in the IML (Bii) and the lamina X (Biii); some were scattered throughout the dorsal laminae (Bi) or the lfu (Bii), and accumulated in the outer rim of the white matter (B, Bii). A few c-Fos positive cells scattered around the cc were observed in the cord incubated at ≤ 10°C (Aiv), which were absent when the cord was incubated at 25°C (Biv). Scale bars = 150 μm in (A, B) and 60 μm in (Ai-iv, Bi-iv).

c-Fos expression patterns in the cords incubated in bath temperatures favorable (25°C) or unfavorable (≤ 10°C) for SND genesis were compared in 7 experiments. Each experiment consisted of two cords incubated for 3 h at ≤ 10°C and 25°C, respectively. c-Fos-positive nuclei were rarely observed in cords incubated at ≤ 10°C (Fig. 3A); only a few were found in the IML (Fig. 3Aii) and the area around the central canal (cc; Fig. 3Aiv). The c-Fos-positive cells in the area around the cc were also observed in cords incubated at 25°C for 1.5 h (Fig. 2A). However, they did not persist in cords incubated at 25°C for 3–6 h (Figs. 2B, 2C, and 3Biv).

In contrast, abundant c-Fos-positive cells were observed in cords incubated at 25°C. The cells were concentrated in lamina X and the IML. Some were scattered in the dorsal laminae with a denser distribution in the superficial layers (Fig. 3B). c-Fos-positive cells also appeared in the white matter, showing a sparse distribution in the lateral funiculus (lfu) and a dense accumulation around its outer rim (Figs. 3Bii). The distribution patterns were similar across different spinal segments.

**Figure 4:**
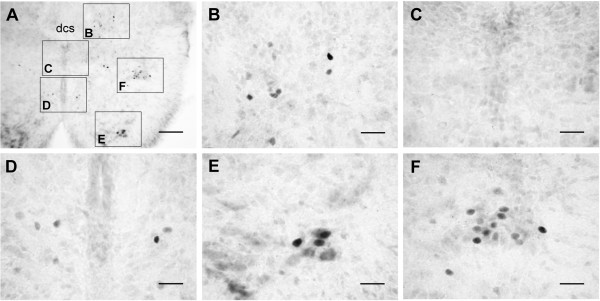
**In vivo c-Fos expression in the cord**. Basal levels of c-Fos expression under normal physiological conditions were promptly preserved by a transcardial perfusion of fixative. (A) Photomicrograph of a transverse section of the T3 spinal cord segment. Insets in (A) are magnified as indicated. c-Fos-positive nuclei were distributed in the dorsal laminae near the dcs (B), around the cc (D), in the ventral horn (E), and in the IML (F). No c-Fos-positive cells were found in lamina X (C), the lfu, or the outer rim of the white matter (A). Scale bars = 100 μm in (A) and 25 μm in (B-F).

For comparisons, in vivo c-Fos expression patterns under physiological conditions were investigated in the rats that were deeply anesthetized with halothane for ~2 min, promptly decerebrated, and transcardially perfused with cold oxygenated aCSF followed by 4% paraformaldehyde (*n *= 7). Without in vitro incubation, the expression of c-Fos under physiological conditions was found in the IML, the dorsal laminae near the dorsal corticospinal tract (dcs), the ventral horn, and areas around the cc (Fig. 4). In vivo c-Fos expression in the ventral horn and the cc areas was not observed in cords with in vitro incubations. In contrast, in vitro c-Fos expression in lamina X and the white matter was not observed in cords in vivo. In the IML and the dorsal laminae, however, the c-Fos expression under in vivo and in vitro conditions was largely similar.

### c-Fos-positive cell identities

**Figure 5:**
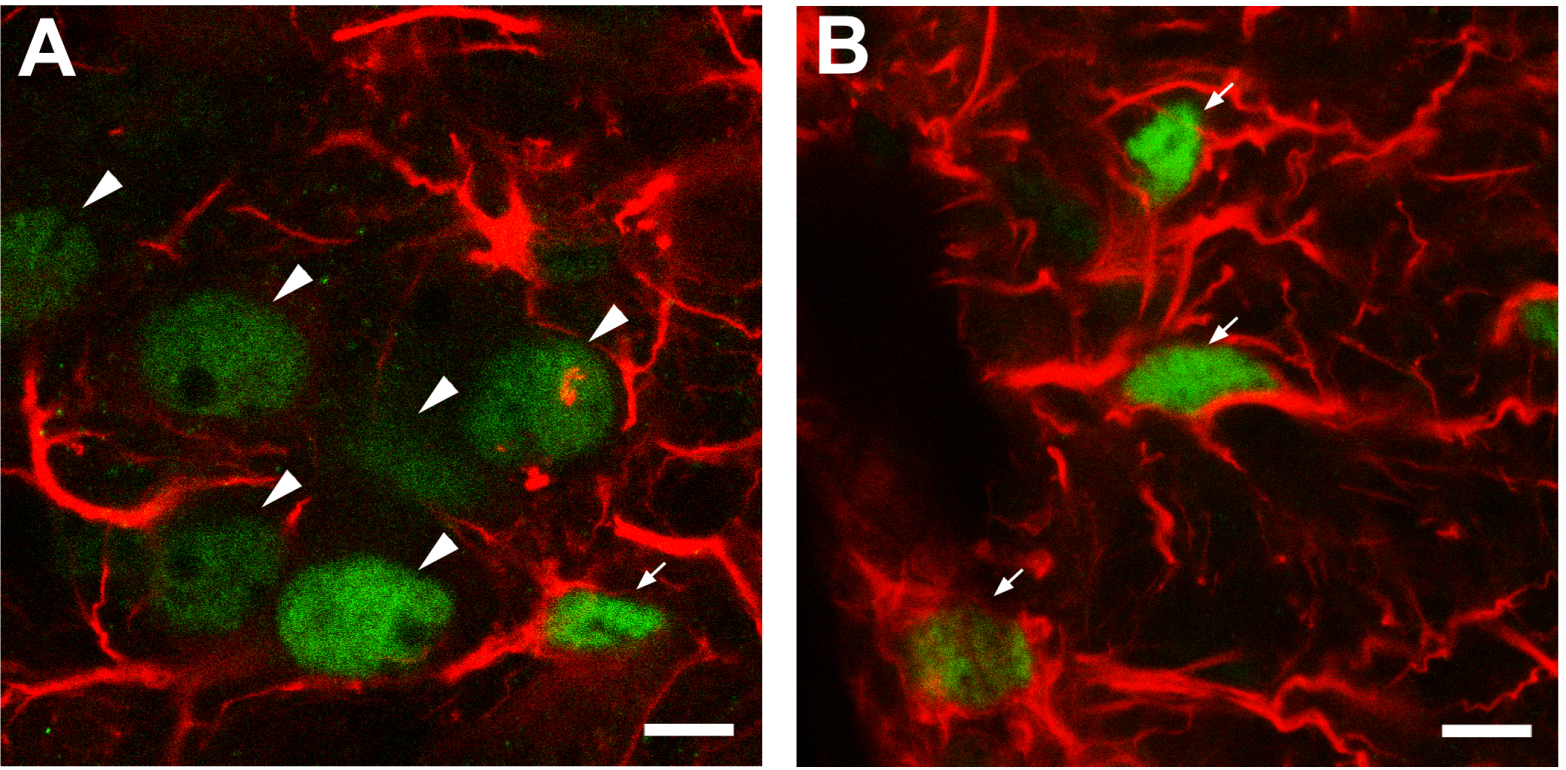
**Confocal images of a transverse section of T6 spinal cord segment, showing c-Fos (FITC) and GFAP (rhodamine) labeling in the IML (*A*) and the lfu (*B*)**. Arrowheads: neuronal nuclei, indicating isolated nuclear c-Fos-IR not surrounded by fibrillary GFAP-IR. Arrows: neuroglial nuclei, indicating nuclear c-Fos-IR in close proximity to fibrillary GFAP-IR. (A) Concentrated c-Fos-positive neurons with large round nuclei (arrowheads) in the IML. The neuroglial cells have small slender nuclei (arrows; one at the upper right is only faintly labeled for c-Fos-IR). (B) c-Fos-positive neuroglial cells in the lfu. Here, nuclear c-Fos-IR is intimately embedded in GFAP-IR (arrows). Scale bars = 10 μm in (A, B).

**Figure 6:**
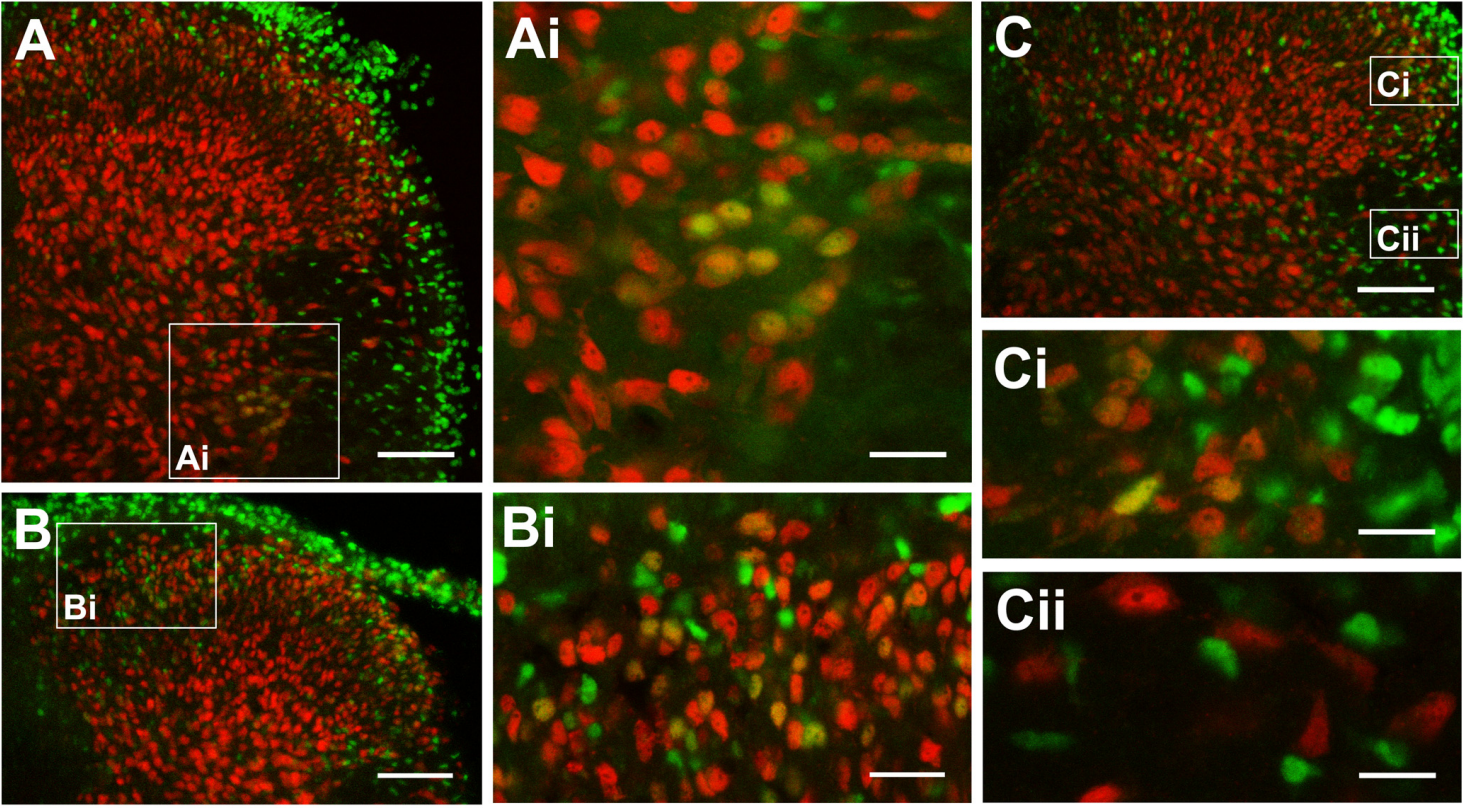
**Distribution of c-Fos-positive neurons in the dorsal laminae and the IML**. Photomicrographs show transverse sections of T3 (A, B) and T9 (C) spinal cord segments. Nuclei with double labeling for c-Fos-IR (FITC) and NeuN-IR (rhodamine) were found in the IML (A, Ai), the internal dorsal laminae (B, Bi) and the external dorsal laminae (C, Ci). All c-Fos-positive nuclei in the lfu and the outer rim of the white matter were NeuN-negative (Cii). Scale bars = 100 μm in (A-C), 35 μm in (Ai, Bi), and 20 μm in (Ci-ii).

The colocalization of c-Fos-IR with IR of glial fibrillary acidic protein (GFAP; *n *= 4) or IR of neuron-specific nuclear protein (NeuN; *n *= 6) was examined to verify whether c-Fos-positive cells were neuroglial cells or neurons. Fibrillary processes with intense GFAP-IR were found in all regions of the cord and more extensively distributed in the white matter than the gray matter. Figure 5 shows the confocal images of the lateral regions of T6 spinal segment double-labeled for c-Fos and GFAP. The large round c-Fos-positive nuclei in the IML were not closely surrounded by GFAP-IR (Fig. 5A). In contrast, the small slender c-Fos-positive nuclei in the lfu were in close proximity to GFAP-IR (Fig. 5B). Figure 6 shows the confocal images of T3 and T9 spinal segments double-labeled for c-Fos and NeuN. NeuN-positive nuclei were mainly distributed in the gray matter. Some were scattered in the lfu (Fig. 6Cii). Nuclei with positive c-Fos-IR and negative NeuN labeling were found in the lfu, the outer rim of the white matter, and lamina X. In contrast, nuclei double-labeled for c-Fos- and NeuN-IR were concentrated in the IML (Fig. 6Ai), and scattered in the dorsal laminae with a denser distribution in the superficial layers (Fig. 6Bi and 6Ci).

**Figure 7:**
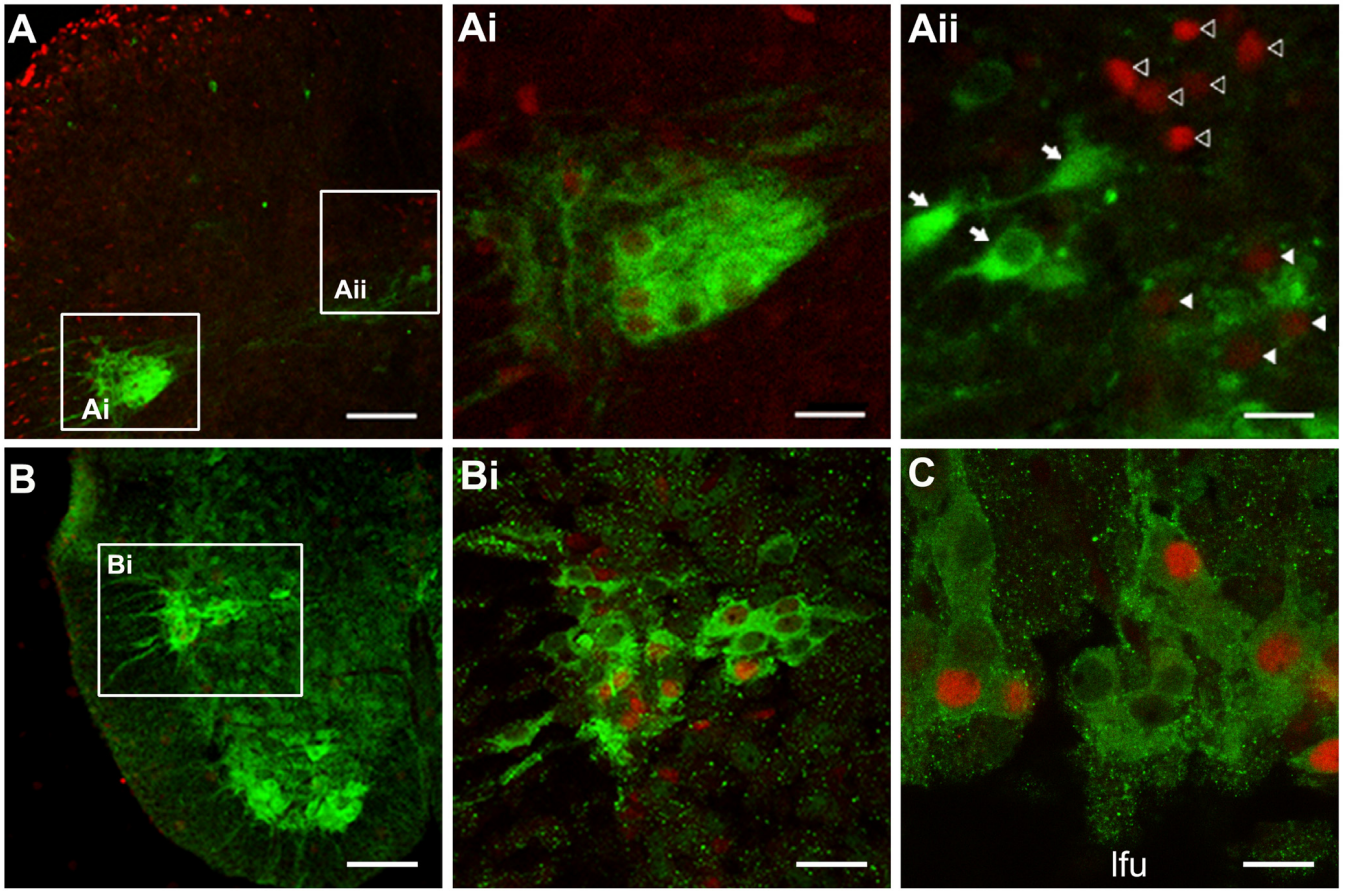
**Colocalization of c-Fos-IR (rhodamine) and ChAT-IR (FITC) to demonstrate that c-Fos-positive neurons in the IML are cholinergic sympathetic preganglionic neurons (SPNs)**. Insets in (A, B) are magnified as indicated. (A) Low magnification photomicrograph of the dorsal quadrant of a transverse section of T3 spinal cord segment. A triangular cluster of IML cells showed intense ChAT-IR that embraced nuclei labeled with c-Fos-IR (Ai). Distribution of ChAT-positive cells extended dorsomedially toward the intermediate zone of lamina X (Aii), where localizations of ChAT-IR and c-Fos-IR were separate. In (Aii), open arrowheads on *Top *indicate c-Fos-positive ChAT-negative cells in the dorsal lamina X; filled arrowheads at *Bottom *indicate nuclei with mild c-Fos-IR surrounded by diffusive ChAT-IR in the ventral lamina X. Arrows indicate ChAT-positive c-Fos-negative cells in the intermediate zone. (B) Low magnification photomicrograph of the ventral quadrant of a transverse section of T5 spinal cord segment. ChAT-IR was present in the IML and the ventral horn; only in the IML, ChAT-IR was colocalized with c-Fos-IR (Bi). (C) High magnification photomicrograph of a horizontal section of T7-T8 spinal cord segments, showing c-Fos-positive nuclei in close proximity to cytoplasmic-like ChAT-IR in the IML. Scale bars = 100 μm in (A, B), 25 μm in (Ai-ii), and 20 μm in (Bi, C).

Because SPNs are one of the cholinergic neurons in the spinal cord, the colocalization of c-Fos-IR with IR of choline acetyltransferase (ChAT) was used as a criterion to identify the histological distribution of active SPNs (*n *= 6). ChAT-IR was mainly observed in the ventral horn, lamina X, intermediomedial region of the gray matter, the IML, and scarcely found in the dorsal laminae (Fig. 7). No c-Fos-positive nuclei were observed in the ventral horn. The c-Fos positive cells distributed in the dorsal lamina X that located just ventral to the dorsal corticospinal tract (dcs) were ChAT-negative (open arrowheads, Fig. 7Aii). Therefore, c-Fos-positive nuclei in close proximity to ChAT-IR were only present in the IML.

### Effects of TTX, MECA, or KYN on c-Fos expression patterns

**Figure 8:**
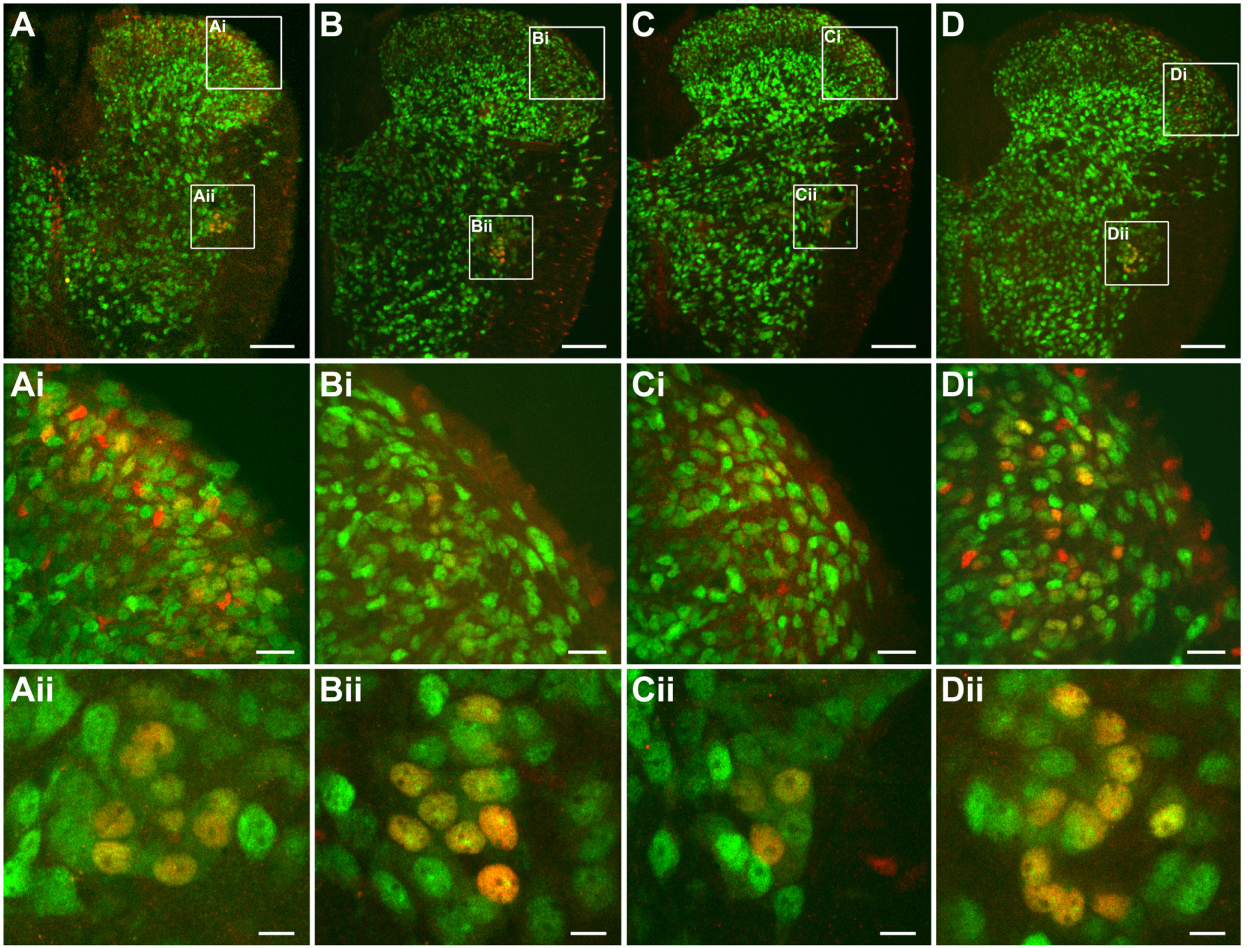
**Distribution of c-Fos-positive neurons in the cords incubated in normal aCSF (A) or aCSF containing 0.5 μM TTX (B), 20 μM MECA (C), or 800 μM KYN (D)**. c-Fos-positive neurons are revealed by double labeling of NeuN-IR (FITC) and c-Fos-IR (rhodamine). Compared with the control (Ai), c-Fos expression in the dorsal laminae neurons was apparently reduced by TTX (Bi) or MECA (Ci) but not by KYN treatment (Di). In the IML, the abundance of c-Fos-positive neurons was diminished only by MECA (Cii), but not by TTX (Bii) or KYN treatment (Dii). Scale bars = 100 μm in (A-D), 20 μm in (Ai-Di), and 10 μm in (Aii-Dii).

**Figure 9:**
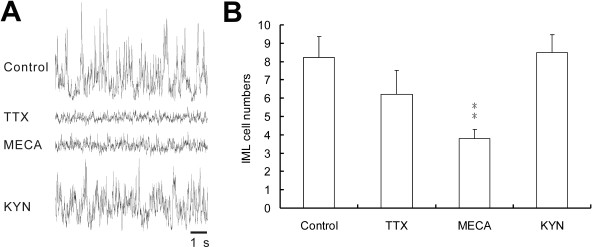
**Drug-induced changes in SND genesis and in the numbers of c-Fos-positive IML neurons**. The spinal cords were incubated in normal aCSF (control) or aCSF containing 0.5 μM TTX, 20 μM MECA, or 800 μM KYN. (A) Original traces of the sSND envelope. sSND was abolished or reduced in cords incubated in aCSF containing TTX or MECA, but not apparently affected by application of KYN. (B) Numbers of c-Fos-positive neurons in the IML. The numbers were obtained from averaging counts of IML cells double labeled for c-Fos-IR and NeuN-IR in transverse sections of T3 spinal cord segments (30-μm-thickness). Compared with the control (8.2 ± 1.1; *n *= 6), the number of c-Fos-positive neurons was significantly reduced by application of MECA (3.8 ± 0.5; *n *= 5; *P* < 0.01), but not by application of TTX (6.2 ± 1.3; *n *= 5) or KYN (8.5 ± 0.6; *n *= 8).

Whether intact synaptic transmission was required for c-Fos expression were examined in cords when SND genesis was interrupted by blocking action potential generation using 0.5 μM TTX or by attenuating endogenous nicotinic receptor activities using 20 μM MECA (*n *= 5 in each test). For comparisons, we also examined c-Fos expression patterns in cords incubated in aCSF containing 800 μM KYN to block ionotropic glutamate receptors (*n *= 8), which did not suppress SND genesis [37]. Figures 8 and 9 illustrate drug-induced change in histological and electrophysiological responses, respectively. sSND is abolished or attenuated by TTX or MECA applications, but not apparently affected by KYN applications (Fig. 9A). In cords incubated in aCSF containing TTX or MECA (Fig. 8Bi and 8Ci), c-Fos-positive neurons were rarely observed in the dorsal laminae; however, they persisted in the IML (Fig. 8Bii and 8Cii). In contrast to cords incubated in aCSF containing 800 μM KYN, c-Fos-positive neurons were observed in the dorsal laminae and the IML (Fig. 8D). Because TTX- or MECA-induced change of c-Fos expression in dorsal laminae was obvious and montage of high-magnification fluorescent photographs were difficult using our image setup, further quantification of c-Fos-positive neurons in dorsal laminae was not pursued. Only the numbers of c-Fos-positive neurons in the IML of T3 spinal cord segments were counted (Fig. 9). Abundance of c-Fos-positive neurons in the IML was not different between cords incubated in normal aCSF and in aCSF containing TTX or KYN. However, c-Fos expression in the IML was significantly reduced in cords incubated in aCSF containing MECA (control vs. MECA: 8.2 ± 1.1 vs. 3.8 ± 0.5; *P *< 0.01).

## Discussion

We have demonstrated that c-Fos-positive neurons were found in the dorsal laminae and the IML when the thoracic spinal cord was incubated under optimal conditions for SND genesis in vitro. The IML neurons were SPNs because they were also ChAT-positive. In cords treated with TTX or MECA to reduce SND genesis, c-Fos expression in the dorsal laminae diminished; however, c-Fos expression in some SPNs persisted. These observations suggest that the excitatory signals originated from action potential-dependent or nicotinic receptor-mediated synaptic transmission are required for c-Fos expression in the dorsal laminae, but not essential for c-Fos expression in some SPNs. Therefore, those SPNs that are capable of c-Fos expression in the presence of TTX or MECA may play key roles in SND genesis.

### c-Fos expression encodes the neural substrates related to SND genesis

The expression of c-Fos and the generation of sympathetic activity in the cord have similar temperature dependence. This temperature effect might indicate only a casual correlation. Thermal effects on biochemical reactions are generic in a biological system. Temperature has profound influences on molecular interactions that govern neurotransmitter release and kinetics of ion channels. Therefore, the c-Fos expression in cords incubated in higher ambient bath temperature could be argued as nonspecific. However, several lines of evidence support the view that the temperature-dependent expression of c-Fos is related to SND genesis. First, in cords kept at the optimal temperature for SND genesis and incubated for 3 h to allow in vitro c-Fos expression, c-Fos-positive cells were found in the IML. This histological observation is consistent with the electrophysiological data that SPNs are active under the in vitro conditions. Second, anatomical and electrophysiological studies have shown that sympathetic-correlated neurons are distributed in the medial dorsal parts of thoracic spinal cord [15-17,21]. Consistent with previous findings, we found that the c-Fos-positive neurons are distributed in the dorsal laminae. The similarity in anatomical distribution indicates that some dorsal laminae neurons may participate in SND genesis in vitro. Third, applications of MECA suppressed SND genesis in cords [36] and reduced c-Fos expression in the dorsal laminae, an effect that was not replicated by applications of KYN. Therefore, c-Fos expression in the dorsal laminae reflects a functional operation of the neural elements underlying SND genesis. The activity of dorsal laminae neurons is likely to be driven, either directly or indirectly, by nicotinic receptor activation.

### Implications of glial activation for in vitro SND genesis

One intriguing observation in this study was that with in vitro incubations, c-Fos-positive and NeuN-negative glial-like cells were abundant in lamina X, the dorsal laminae, and the lfu. c-Fos expression in these areas was largely absent under in vivo conditions (Fig. 4). The lack of blood circulation under in vitro conditions inevitably led to interstitial accumulations of metabolites, which might result in glial activation as revealed by c-Fos expression. Glial activation is required to maintain a homeostatic neuronal environment [44-46]. Therefore, the distribution pattern of c-Fos-positive neuroglia implies a spontaneous neural activity in the regions. Whether the interplay between the active neural elements and the activated neuroglia plays a role in SND genesis awaits for further studies.

### Spontaneously active SPNs in the IML

Only the cholinergic neurons aggregated in the IML expressed c-Fos. All the other cholinergic neurons were c-Fos-negative. In addition to the IML, SPNs are distributed in the intermediomedial regions and the central autonomic areas. The lack of c-Fos expression in these regions suggests that these SPNs do not contribute to SND genesis in vitro. Therefore, the SPNs of the IML are the only active cholinergic neurons during SND genesis in vitro.

Surprisingly, c-Fos expression in the SPNs of the IML was largely not altered after blocking action potential generation by TTX applications, but it was significantly reduced after blocking endogenous nicotinic receptor activities by MECA applications. It has been reported that nicotine-activated c-Fos expression in PC12 cells is TTX-resistant but MECA-sensitive [47]. TTX-resistant c-Fos expression was also observed in chemosensitive cells in the superficial ventral medulla of rats [48]. In our studies, although the suppression of SND genesis induced by TTX or MECA applications was similar, its underlying mechanisms were different. TTX applications block action potential-dependent neurotransmission and leave many TTX-resistant channel activities intact. In contrast, MECA applications block nicotinic receptor-mediated neurotransmission and let the neurotransmissions mediated by other receptor types function normally. SPNs are inhibited by GABAergic and glycinergic neurotransmission [23,49-51]. These inhibitory activities would not be directly affected by MECA, but could be abolished by TTX. It is likely that, impinged upon SPNs, the presence of inhibitory activities during MECA applications explains why c-Fos expression is reduced by MECA but not by TTX.

Moreover, MECA is an open-channel blocker, which can only be effective when nicotinic receptors are activated [52]. Thus, MECA-induced suppression of c-Fos expression in the IML is partly because these SPNs are continuously excited, be it directly or indirectly, by nicotinic receptor activation [36]. The source of cholinergic activity for nicotinic receptor activation was not determined in this study. However, colocalization of c-Fos-IR with ChAT-IR is only found in the IML (Fig. 7), suggesting that IML SPNs are the only active cholinergic neurons in the cord. Therefore, under our experimental conditions, initiation of SND may originate from a few spontaneously active SPNs in the IML, which are capable of TTX- or MECA-resistant c-Fos expression. This view is consistent with earlier observations from other laboratories that some SPNs are spontaneously active [7,8,53].

### Intraspinal sources for induction of c-Fos expression in dorsal laminae

Sympathetic-correlated neurons are mainly located in the dorsal laminae I-V [21,22,54,55], lamina VII [19], and in the vicinity of IML [56,57]. Under normal physiological conditions, supraspinal commands exert inhibitory influences to suppress sympathetic-correlated neurons in the dorsal laminae, which may normally function as relays for spinal sympathetic reflex [58-61].

We have found that, in addition to the presence of active SPNs in the IML, active neurons double-labeled for c-Fos and NeuN are also present in the dorsal laminae (Fig. 6). This finding is similar to the observations in adult rats that c-Fos expression after spinal cord transection is found in laminae I, IIo (outer substantia gelatinosa), Vre (lateral reticulated division), VII (lamina intermedia), X, and the IML [62]. Induction of c-Fos expression in dorsal laminae neurons could not be attributed to the excitatory signals from extraspinal sources. First, the brain stem and the cervical cord were removed in this nerve-cord preparation. Second, somatovisceral inputs from the primary afferents were quiescent under in vitro conditions, as indicated by the absence of c-Fos-positive cells in the dorsal root ganglion. Therefore, an intraspinal source is responsible for the induction of c-Fos expression in the dorsal laminae.

A few SPNs in vertebrates have intraspinal axon collaterals [9,63,64]. In adult rats, some SPNs with somata located outside the IML have axons branched within the spinal gray [63]. The axon collateral terminals are exclusively presynaptic to small caliber dendrites and form only asymmetric specializations, suggesting a conveyance of excitatory signals [63,64]. In neonatal rats, histological examinations of 45 SPNs reveal that 2 SPNs have axonal branches in lamina VII or the ventral horn [9]. Through their axon collaterals, we are tempted to speculate that some SPNs may play a stimulatory role in activation of other neural groups in the cord, including the sympathetic-correlated neurons in the dorsal laminae. Our observation that c-Fos expression in the dorsal laminae neurons was almost abolished by incubating the cords in aCSF containing TTX or MECA lends limited support to this speculation.

## Abbreviations

cc: central canal; ChAT: choline acetyltransferase; DAB: diaminobenzidine; dcs: dorsal corticospinal tract; FITC: fluorescein isothiocyanate; GFAP: glial fibrillary acidic protein; IML: intermediolateral cell column; IR: immunoreactivity; KYN: kynurenate; lfu: lateral funiculus; MECA: mecamylamine; NeuN: neuron-specific nuclear protein; SND: sympathetic nerve discharge; SPN: sympathetic preganglionic neuron; TTX: tetrodotoxin.

## Competing interests

The authors declare that they have no competing interests.

## Authors' contributions

CKS and CMH participated in experimental design, data interpretation, and manuscript preparation. HHK and YCW conducted pharmacological and immunohistochemical experiments and data analysis. CYC was involved in overall research planning and assisted in manuscript preparation. All authors read and approved the final manuscript.
